# Isolation and Characterization of Polyacrylamide-Degrading Bacteria from Dewatered Sludge

**DOI:** 10.3390/ijerph120404214

**Published:** 2015-04-16

**Authors:** Feng Yu, Ruimin Fu, Yun Xie, Wuling Chen

**Affiliations:** 1 College of Life Sciences, Northwest University, Xi’an 710021, China; E-Mails: yufeng_029@163.com (F.Y.); angelaminmin@163.com (R.F.); 2 Test Department, Northwest Maternal and Child Hospital, Xi’an 710061, China; E-Mail: lovenight029@gmail.com

**Keywords:** polyacrylamide, biodegradation, dewatered sludge, microorganisms

## Abstract

Polyacrylamide (PAM) is a water-soluble polymer that is widely used as a flocculant in sewage treatment. The accumulation of PAM affects the formation of dewatered sludge and potentially produces hazardous monomers. In the present study, the bacterial strain HI47 was isolated from dewatered sludge. This strain could metabolize PAM as its sole nutrient source and was subsequently identified as *Pseudomonas putida*. The efficiency of PAM degradation was 31.1% in 7 days and exceeded 45% under optimum culture condition (pH 7.2, 39 °C and 100 rpm). The addition of yeast extract and glucose improved the bacterial growth and PAM degradation. The degraded PAM samples were analyzed by gel-filtration chromatography, Fourier transform infrared and high-performance liquid chromatography. The results showed that high-molecular-weight PAM was partly cleaved to small molecular oligomer derivatives and part of the amide groups of PAM had been converted to carboxyl groups. The biodegradation did not accumulate acrylamide monomers. Based on the SDS-PAGE and *N*-terminal sequencing results, the PAM amide groups were converted into carboxyl groups by a PAM-induced extracellular enzyme from the aliphatic amidase family.

## 1. Introduction

Polyacrylamide (PAM) is a water-soluble polymer that is usually produced through the polymerization of acrylamide with one or more copolymers. The amide groups of PAM form hydrogen bonds in aqueous solutions, and high-molecular-weight PAM is an effective flocculant of suspended solids in water via charge neutralization and interparticle bridging [1]. Thus, PAM is commonly used as a flocculant to improve sedimentation in sewage treatment [2], oil recovery [3], paper manufacturing [4], mining, and other fields [5,6,7]. China is the world’s largest consumer of PAM. Nearly 330,000 t of PAM was consumed in 2008, which was approximately 38% of the world's total consumption. Oil recovery accounted for 57% of PAM consumption, and sewage treatment accounted for 21% [8]. During the sewage treatment process, PAM is mostly consumed during excess sludge dewatering and ultimately accumulates in dewatered sludge. Although sludge dewatered with PAM has a low water content, the sludge often remains humid and tends to form clumps. This coagulated sludge is difficult to dispense and can increase the cost of sludge drying [9]. Various reports have indicated that commercial non-toxic PAM can be degraded into hazardous acrylamide monomers under the influence of specific physical and chemical factors [10,11]. Given that residual PAM in sludge degrades slowly under natural conditions, the improper disposal ways of sludge like landfill and land application may discharge of PAM and acrylamide monomer into terrestrial and aquatic ecosystems. It can form hemoglobin adducts and induce abnormalities in the daughter cells of animals and plants [12]. According to China’s 12th five-year plan, the urban sewage treatment rate exceeded 70% in 2010 and is expected to reach 85% in the next five years. As the country gradually increases its efforts to protect the environment, PAM consumption in sewage treatment will definitely increase. Therefore, a safe and economical transformation of residual PAM should be studied.

The microbial biodegradation of PAM has long been studied. Previous studies suggested that microorganisms are able to degraded and utilized PAM as a nitrogen source in both aerobic and anaerobic environments [13,14]. Microorganisms produce a polyacrylamide induced amidase to deaminate PAM [15]. Few studies showed that bacteria can utilize or partly utilize PAM as a carbon source [16,17]. Several strains that partially use hydrolyzed PAM as the nitrogen and carbon source was also discovered [18,19]. It showed that the carbon backbone chain of PAM can be broken down to form volatile fatty acids that function as carbon sources under anaerobic conditions [1]. Although experimental evidence has achieved some level of success, further research is still necessary. The present paper aimed to screen for a PAM-degrading microorganism from dewatered sludge and evaluated the effect of available nutrients on bacterial growth and degradation activity. Specific bacterial characteristics during degradation were also explored.

## 2. Materials and Methods

### 2.1. Samples

Sludge samples were collected from two sewage plants. One is located in northern suburb of Xi’an City and the other in Shoushan town, Mei County. The molecular weight of PAM used to dewater the excess sludge was approximately 0.5 × 10^7^ Da–1.6 × 10^7^ Da in these sewage plants. Microorganisms were isolated from dewatered sludge that had been accumulated and exposed to light for more than 20 days. Tweezers were used to break the hardened sludge surface. The moisture part under surface was sampled. Samples were kept in sterile wide-mouth bottles at 4 °C.

### 2.2. Media

The amount of PAM adds in sludge is about 3 to7 Kg per ton of dry sludge according to different sewage treatment plant. Actually, the concentration of residual PAM in dewatered sludge is lower than addition dosage. Given that calculation methods of PAM concentration are different in water and sludge, we add PAM at the concentration of 1 g·L^−1^ in experiments. The PAM applied in the experiments was supplied by the Tuopu Water Purification Materials Co., Ltd. (Gongyi, China). Its molecular weight was approximately 1.7 × 10^7^ Da‒2.2 × 10^7^ Da. Two kinds of culture media were used. The polymer medium contained PAM as the only nutrient for the selection of PAM-degrading bacteria. The second medium was a nutrient medium for bacterial enrichment. The polymer medium was based on mineral salt medium with 0.5 g·L^−1^ sodium chloride, 0.1 g·L^−1^ calcium chloride, 0.25 g·L^−1^ magnesium sulfate, 0.5 g·L^−1^ sodium dihydrogen phosphate, 1.0 g·L^−1^ dipotassium phosphate, and 1 g·L^−1^ PAM at pH 7.2‒7.4 [20]. The nutrient medium was based on mineral salt medium supplemented with 5.0 g·L^−1^ yeast extract, 10.0 g·L^−1^ peptone, and 5.0 g·L^−1^ sodium chloride at pH 7.2‒7.4 [18]. All chemical reagents were bought from Sigma-Aldrich Company (St. Louis., MO, USA). Media were sterilized at 121 °C for 20 min before use.

### 2.3. Isolation of Strains

Each culture flask contained 10 g of the sludge samples dissolved in 150 mL of the liquid nutrient medium. The cultures were incubated aerobically on a shaking platform at 80 rpm–100 rpm and 30 °C for 2 days. Subsequently, 10 mL of the initial culture was used to inoculate 150 mL of the liquid polymer medium and the cultures were incubated at 30 °C for 5 days. Each enrichment period lasted for 7 days of cultivation. At the end of each period, 10 mL of the enrichment culture was seeded into fresh liquid polymer medium and incubated for another 7 days. The cultivation process was performed for at least four periods.

After a month of cultivation, the broth cultures were streaked onto nutrient agar plates and incubated aerobically at 30 °C. When colonies emerged on the plates, different strains were selected and separately inoculated into the polymer medium such that PAM was the sole nutrient source. The strain was inoculated in 150 mL of the liquid polymer medium which prior sterilized at 121 °C for 20 min. The culture was incubated aerobically at 100 rpm and 30 °C for PAM degradation tests. The cell concentration and PAM degradation ability were evaluated during the cultivation. The microbial biomass was measured by spread plate method using culture flasks. Growth curve was performed by measuring the optical density (OD) at 660 nm and transferred to cfu (colony forming unit) through calibration curve. The PAM concentration was measured by the starch-cadmium iodide method [21]. The PAM removal efficiency was calculated according to the following formula: *R* = (*Q*_0_ − *Q*_1_)/*Q*_0_, where *Q*_0_ and *Q*_1_ represent the PAM concentration before and after degradation, respectively. A flask of PAM medium without inoculation was set as the blank test to avoid the influence of PAM shearing, autoclaving, and self-degradation in the experiment. Strains that showed good performance in their PAM removal efficiency were chosen for further study.

### 2.4. Identification of Isolates

The chosen strain was identified by an automated bacterial identification system and its 16S rDNA sequence. Metabolite identification was conducted with the bioMérieux VITEK 2 system, which is an automated microbiology system that utilizes a growth-based technology. The colonies in pure culture were transferred and suspended in 3.0 mL of 0.45% sterile saline. The turbidity of the bacterial suspensions was adjusted with a McFarland nephelometer to fit the card inoculation. The GN card was selected as the reagent card. Each reagent card was inoculated with the microbial suspensions using an integrated vacuum apparatus. The test took 6 h–10 h [22].

The 16S rDNA analysis was conducted after automated identification to further confirm the results. The total DNA of the bacterial isolate was extracted with a TaKaRa bacteria genomic DNA extraction kit. The universal primers 8F (5ʹ-AGAGTTTGATCCTGGCTCAG-3ʹ) and 1492R (5ʹ-TACG-GTTACCTTGTTACGACTT-3ʹ) were applied for PCR amplification [23]. The 20 μL PCR mixture contained 2 μL of the PCR buffer (20 mM MgCl_2_), 0.2 mM of each dNTP, 0.2 μL of *Taq* DNA polymerase, 0.5 μL of each primer (10 µmol·μL^−1^), and 1 μL of the DNA template. All the reagents in the PCR system were supplied by Sangon Co., Ltd. (Shanghai, China). Amplification was performed with the initial denaturation at 94 °C for 5 min, followed by 30 cycles of 94 °C for 30 s, 55 °C for 60 s, and 72 °C for 70 s, and a final extension at 72 °C for 10 min. The PCR products (approximately 1500 bp) were purified and detected by agarose gel electrophoresis. Purified amplicons were inserted into the pMD18-T vector (TaKaRa Biotechnology, Dalian, China) and used to transform *Escherichia coli* DH5α. The 16S rDNA recombinant plasmid was extracted and sequenced by the Beijing Sunbiotech Co., Ltd. (Beijing, China). The sequence was submitted and analyzed with the BLAST tools (National Center for Biotechnology Information databases) to identify the bacterial isolate.

### 2.5. Determination of PAM Degradation

The samples were taken from cultures, which were incubated in the liquid polymer media at 30 °C under shaking (100 rpm) for 7 days. Uninoculated media were set as control. The changes of PAM molecular weights (MW) after degradation were determined by gel-filtration chromatography (GFC). Samples were analyzed with an LC-10A apparatus (Shimadzu, Kyoto, Japan) equipped with an Ultrahydrogel Column 1000 (12 µm, 300 mm × 7.8 mm) and an RID-10A differential detector. Samples were filtered by filterable membranes (pore size, 0.45 µm) prior to analysis. The GFC was run with the 0.1 M sodium nitrate solution at a flow rate of 0.5 mL/min.

Fourier transform infrared (FT-IR) spectroscopy was applied to analyze the side chain functional groups changes in the medium before and after degradation. Samples were extracted by placing each into a separating funnel with 5% petroleum ether (volume fraction). Polymers in the lower layer were filtered by membranes (pore size, 0.45 µm) and purified with methanol. After drying at 60 °C, the purified polymer samples were dispersed into KBr pellets. The samples were analyzed with an Equinox 55 FT-IR spectrometer (Bruker, Ettlingen, Germany) at a scanning range of 4000 cm^−1^–500 cm^−1^.

Samples after biodegradation were monitored by HPLC to verify if the acrylamide monomer had accumulated. Samples were filtered by filterable membranes (pore size, 0.45 µm) prior to HPLC analysis. Samples were analyzed with a Delta 600 apparatus (Waters, Milford, MA, USA) that was equipped with a Spherisorb ODS-1 column (150 mm × 4.6 mm, 5 μm). A methanol-water (55:45) mixture was used as the mobile phase. The flow rate was 0.5 mL·min^−1^, and the injection volume was 20 μL. The detection wavelength was 210 nm, at which PAM and acrylamide monomer have maximum absorption [24].

### 2.6. Extracellular Enzyme in PAM Degradation

The activated strain was seeded in the 150 mL liquid polymer medium. Samples were taken from the culture every 12 h. Each sample was centrifuged at 4000 rpm (1700× *g*) for 30 min to collect the extracellular matrix. The supernatant was subsequently filtered through a cellulose acetate membrane (pore size, 0.2 µm). Proteins in supernatant were extracted by the ammonium sulfate precipitation method [25]. Ammonium sulfate was added to the supernatant to a final concentration of 70%. The extract was then centrifuged at 10,000 rpm (11,000× *g*) for 30 min at 4 °C. The obtained precipitate was dissolved with PBS and then dialyzed against double-distilled water at 4 °C overnight. The protein concentration was determined by the Bradford method [26]. The standard solution of 1 mg·mL^−1^ bovine serum albumin was prepared to set the standard absorbance curve. Crude proteins were separated by the running supernatant on 8% SDS-PAGE at a constant voltage of 80 V with the mini-PROTEAN electrophoresis system (Bio-Rad, Hercules, CA, USA). After the proteins were separated, the gel was stained with Coomassie Brilliant Blue R-250. The stained proteins were transferred onto a PVDF membrane by a semi-dry transfer apparatus containing the transfer buffer (48 mM Tris, 39 mM glycine, 0.2% SDS, and 20% methanol, with water to a 1 L volume); a constant current of 0.8 mA·cm^−2^ was applied for 90 min to facilitate the transfer [27]. The blotted bands on the PVDF membrane were cut and subjected to *N*-terminal sequencing by the Shanghai GeneCore BioTechnologies Co., Ltd. (Shanghai, China).

## 3. Results and Discussion

### 3.1. Bacteria Isolation and Identification

**Figure 1:**
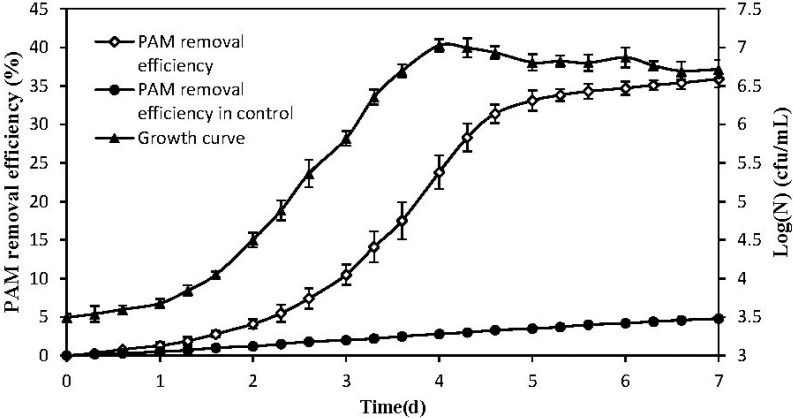
Time course of growth curve and PAM removal efficiency of *P. putida* HI47.

**Figure 2:**
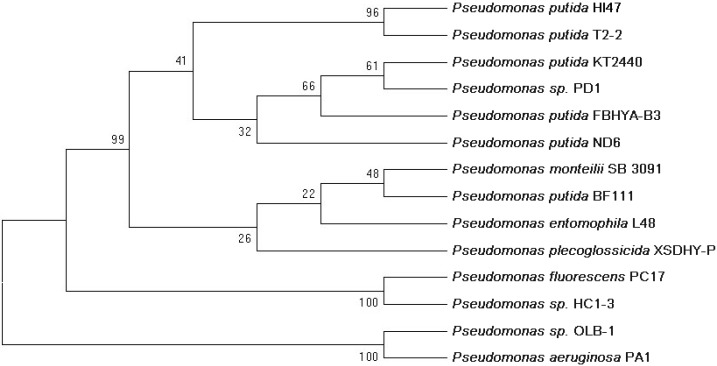
The phylogenetic tree of *Pseudomonas* strain homologues to the HI47. The phylogenetic tree was constructed by the neighbor-joining method with MEGA 5.0. Bootstrap value is set to 1000. Species names are followed by the accession numbers of their 16S rRNA gene sequences.

Seven strains that could grow on the polymer medium were isolated from the sludge samples during the preliminary screening. The selected strain HI47 exhibited the highest removal efficiency such that it degraded 31.1% PAM after 7 d of cultivation in despite of PAM self-degradation (Figure 1). The VITEK 2 results (Table A1) showed with high confidence levels that the HI47 strain corresponded to *Pseudomonas putida* with 90% probability. The 16S rDNA of the bacteria was amplified and sequenced. The BLAST search results indicated that the isolate was consistently 99% similar to *P. putida*. The construction of a phylogenetic tree using the neighbor-joining method (Figure 2) demonstrated a close relationship between the HI47 (KJ820740) isolate and *P. putida* in support of the VITEK 2 results.

*P. putida* has been exploited for environmental bioremediation and biodegradation in several studies [28,29,30]. This species has been applied in xenobiotics [31,32] and polymer degradation [33]. However, its role in PAM degradation has not been reported. Thus, to our knowledge, this paper is the first to describe the degradation of PAM by *P. putida*.

**Figure 3:**
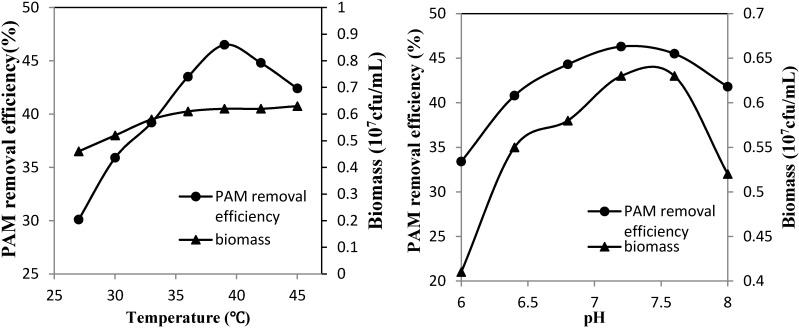
Effect of temperature (**a**) and pH (**b**) on PAM removal efficiency and viable cell biomass.

The effect of temperature and pH on PAM degradation was explored. As shown in Figure 3, the PAM removal efficiency was improved with increasing incubation temperature. This efficiency reached its maximum at 46.5% when the strain was cultured at 39 °C. Although the PAM removal efficiency started to decrease at temperatures more than 40 °C, the bacteria growth remained unaffected. The biomass continued to grow even when temperatures exceeded 45 °C. In addition, the optimum pH of PAM degradation was approximately 7.2. High levels of degradation could be maintained over 40% when the pH was between 6.4 and 8.0. The optimum pH for bacterial growth ranged from 7.2 to 7.6. Therefore, the optimum conditions of PAM degradation and bacterial growth were not entirely consistent. The isolated strain was more sensitive to the hydrogen ion concentration, whereas PAM degradation was restricted by the reaction temperature. The results indicated that the extracellular enzyme was involved in degradation.

**Figure 4:**
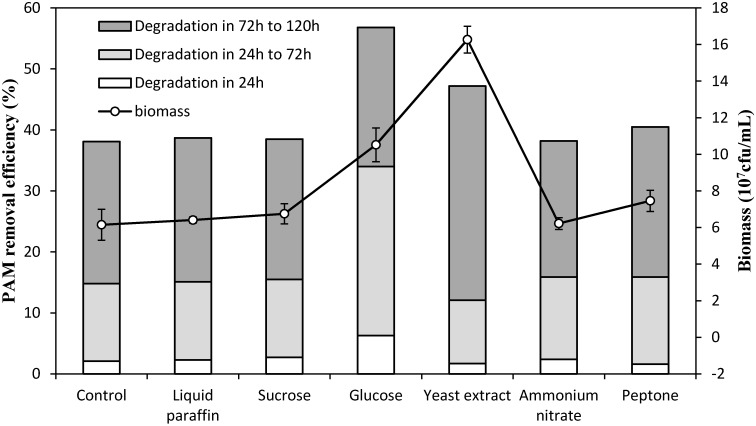
Effect of exogenous nutrients on PAM removal efficiency and viable cell biomass in 5 days of cultivation.

Previous studies showed that bacteria hydrolyze the PAM-substituted amides to release ammonia as the nitrogen source [34]. Several research groups have observed that additional carbon sources can promote bacterial growth and PAM degradation [18,19]. To investigate the effect of nutrient availability, we supplemented the polymer medium with 200 mg·L^−1^ exogenous nutrients and determined the resulting biomass and PAM concentration. As shown in Figure 4, inorganic nitrogen, liquid paraffin and sucrose merely contribute to the PAM degradation and biomass as compared with the control (medium without additional nutrients). According to Duncan’s multiple range test, PAM removal efficiency of groups added glucose, yeast extract and peptone were significantly different at *p* = 0.05. Yeast extract significantly promoted cell growth to 1.63 × 10^8^ cfu·mL^−1^. It also increased PAM degradation to 47.2% in 5 days. The efficiency of PAM degradation was further improved to 56.8% when glucose was present in the culture medium. However, less biomass was obtained with glucose than when yeast extract was added to the culture medium. This trend may be explained by the fact that glucose can only be used as a carbon source. The PAM degradation rate in different period showed that yeast extract was metabolized prior to PAM as source of organic carbon and nitrogen in bacterial growth. The initiation of PAM degradation in the culture especially after 72 h may be attributed to the depletion of yeast extract to sustain the number of viable cells. In the case of glucose, cell growth was restricted by the limited availability of a nitrogen source. The isolated strain was stimulated to degrade PAM to acquire the required nitrogen for growth. This result was not discussed in previous studies.

### 3.2. Determination of PAM Degradation

**Figure 5:**
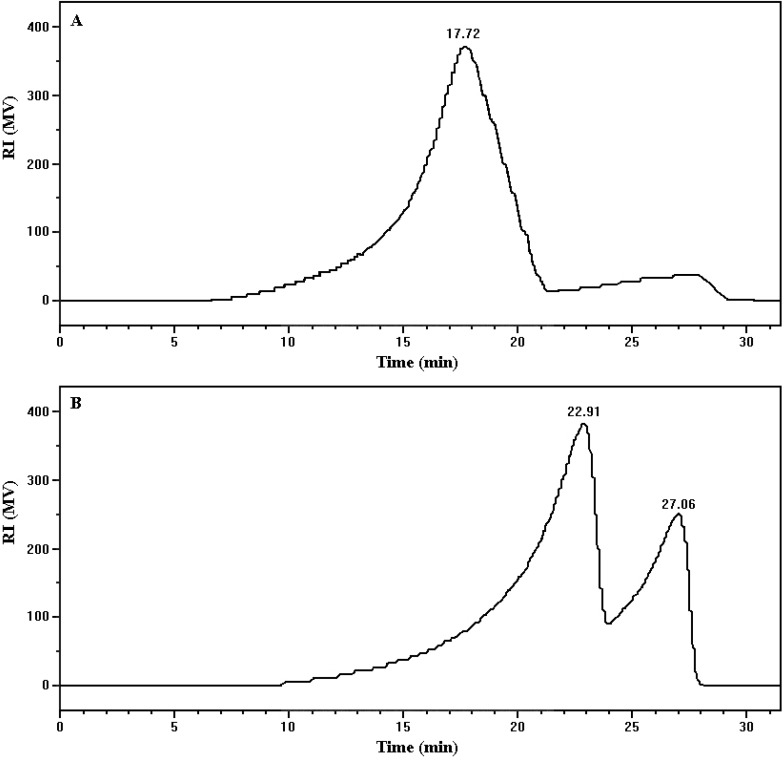
GPC analysis of PAM before and after biodegradation. (**A**) Chromatography of uninoculated control; (**B)** chromatography of PAM after 7 days biodegradation.

The MW (molecular weight) distributions of PAM before and after cultivation were investigated by GFC to study whether the cleavage of PAM backbone chain exists in the process of PAM utilized as sole nutrient. As shown in Figure 5, the peak with retention time of 17.72 min appeared in uninoculated control. In the sample after biodegradation, two peaks with retention times of 22.91 min and 27.06 min were found. The transfer of peaks show higher-molecular-weight polymer was cleaved into small molecular oligomer derivatives, which are more suitable for microbial consumption. It indicates the breaking of carbon–carbon bonds is associated with biodegradation of PAM.

**Figure 6:**
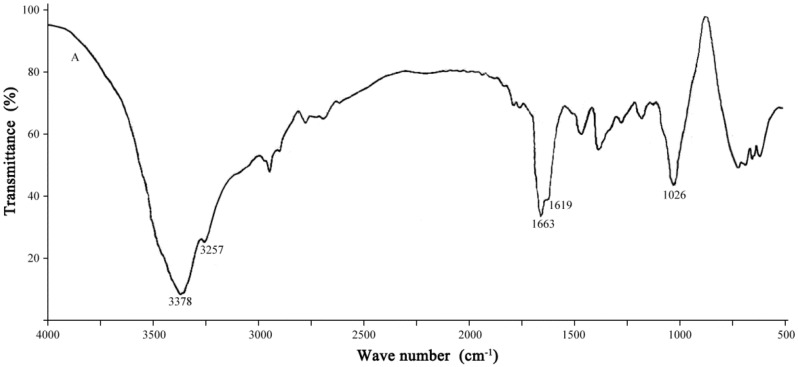

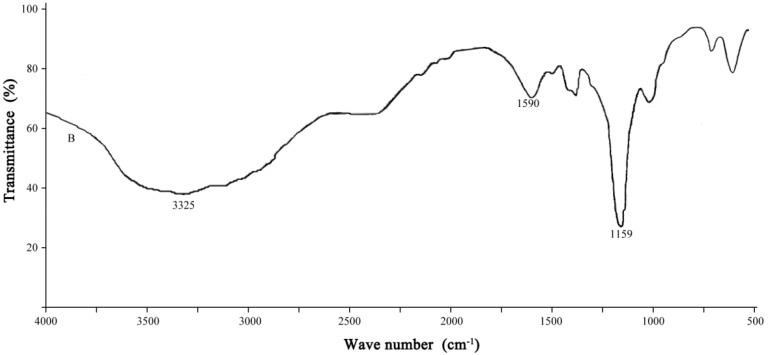
FT-IR analysis of PAM before and after biodegradation. Line **A** is the spectrum of uninoculated control; line **B** is the spectrum of PAM after 7 days biodegradation.

The samples incubated after 7 days were analyzed by infrared spectroscopy to investigate functional groups. The changes of functional groups were determined by comparing the absorption peaks before and after degradation. Line A in Figure 6 is the absorbance spectra for the undegraded PAM control, and Line B is the absorbance spectra of PAM after degradation. Line A possesses the typical characteristics of PAM. The absorbance peaks at 3378 and 3257 cm^−1^ represent amidogen. The absorbance peaks at 1663, 1619, and 1026 cm^−1^ correspond to the C=O stretching vibration, N–H bending vibration, and C–N bond, respectively. The peak at 3378 cm^−1^ was replaced by a broad absorption peak in Line B, which indicated that the amidogen level had decreased. The peak at 1159 cm^−1^ is the C–O stretching vibration. The peak at 1663 cm^−1^ in Line A disappeared and became a weak peak at 1590 cm^−1^ in Line B, which indicated that the amide group was converted into a carboxyl group. The observed spectra indicated that part of the amide groups in the samples were hydrolyzed and converted into carboxyl groups after bacterial degradation. These results are consistent with previous results reported by Wen *et al.* [19].

**Figure 7:**
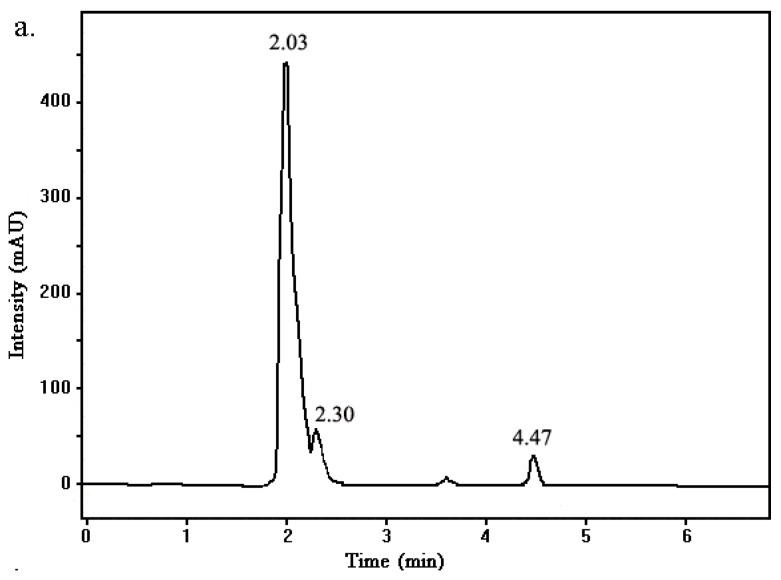

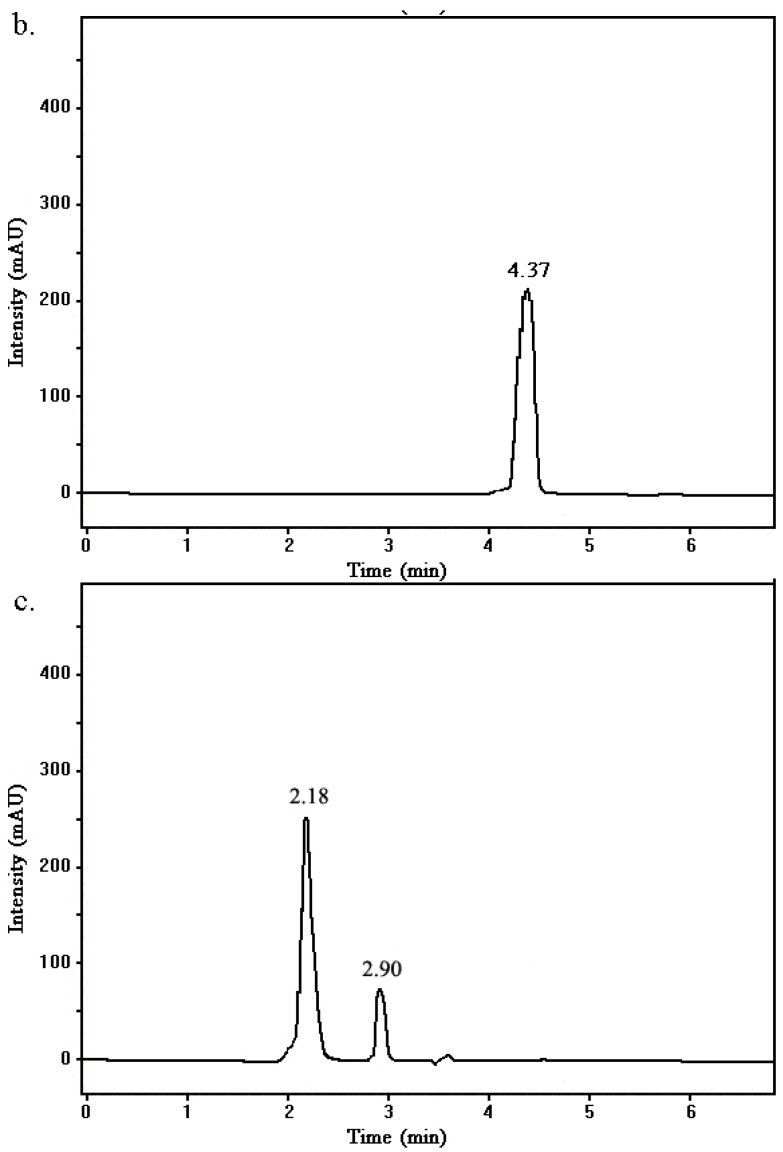
HPLC analysis of PAM samples and acrylamide monomer. (**a**) undegraded PAM sample; (**b**) pure acrylamide monomer; (**c**) PAM sample after biodegradation.

The PAM samples before and after biodegradation were analyzed to determine whether the acrylamide monomer exists after biodegradation. The HPLC profiles of PAM before and after biodegradation are shown in Figure 7. Three peaks appear in the liquid chromatogram of the uninoculated control sample, with respective retention times of 2.03, 2.30, and 4.47 min (Figure 7a). PAM had acrylamide monomer residues in the sample because of self-degradation. The peak corresponding to a pure acrylamide sample was observed after 4 min (Figure 7b). After 7 days of biodegradation, only two peaks with retention times of 2.18 and 2.90 min (Figure 7c) remained in the PAM sample. The area of main peak was reduced, and the peak of acrylamide disappeared. These results show that PAM biodegradation did not cause the accumulation of acrylamide monomers. However, an analysis of the degradation product was not performed in our experiments. Furthermore, other metabolites appeared during degradation; these products need further exploration in future studies.

### 3.3. Determination of the Extracellular Enzyme

**Figure 8:**
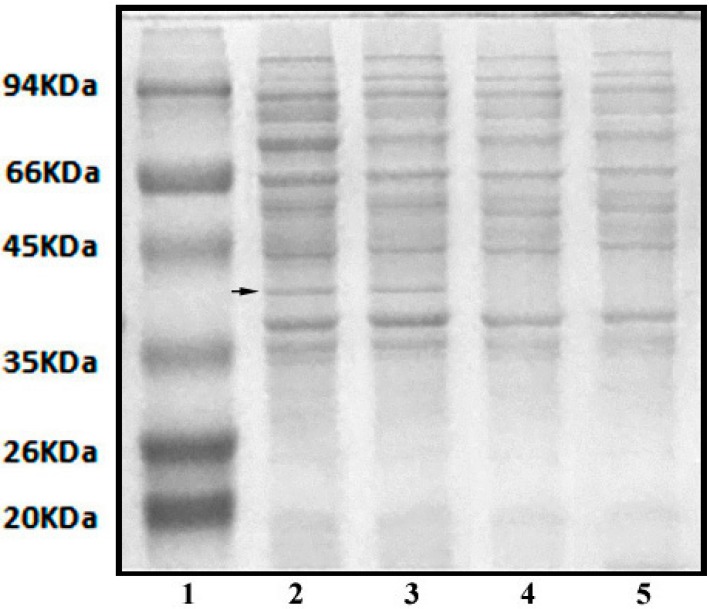
SDS-PAGE analysis of extracellular proteins. Lane **1**: middle mw protein marker; Lane **2**–**5**: extract of extracellular proteins from bacteria incubated in polymer medium for 36 h, 24 h, 12 h and 0 h.

Previous studies noted that a PAM-specific amidase is produced during cell growth when PAM is supplied as the substrate. The activity of intracellular amidase is low and relatively constant, whereas the specific extracellular enzyme activity is significantly increased during the period of logarithmic cell growth [15]. High-molecular-weight polymers cannot be transferred through the cell membrane. Thus, enzymes are secreted to the extracellular space to degrade PAM into small molecules. We extracted the extracellular proteins present in the cultures containing PAM. The extracellular proteins were collected and separated via SDS-PAGE. As shown in Figure 8, a protein band between 35 and 45 kDa appeared after 24 h, which suggested that this band was a PAM-induced enzyme. The *N*-terminal sequencing analysis showed that the first 20 *N*-terminal amino acids of the identified protein were VGVAVVNYKM-PRLHTAAEVL. The BLAST search results identified the sequence to be the part of the aliphatic amidase (cd07565). It belongs to the nitrilase superfamily (cl11424), which contains hydrolases that break carbon–nitrogen bonds. This result indicates that the induced enzyme corresponded to the aliphatic amidase family, which exists in different species, such as *Helicobacter*, *Rhodococcus*, and *Enterobacter* [12,35,36]. Aliphatic amidase catalyzes the hydrolysis of middle- or short-chain aliphatic amides and converts them to their organic acids. Several studies suggested that aliphatic amidase can degrade acrylamide. Syed *et al.* [37] purified amidase from the acrylamide-degrading *Burkholderia* sp. DR.Y27 strain, which can degrade a series of short-chain aliphatic amides, including acrylamide, acetamide, and propionamide. Skouloubris *et al.* [38] identified an aliphatic amidase in *Helicobacter pylori* that has propionamide, acrylamide, and acetamide as its ideal substrates. The HPLC analysis results verified that acrylamide did not significantly accumulate after PAM biodegradation and indicated that the specific extracellular amidase had degraded acrylamide. This trend is consistent with the results of Kay-Shoemake *et al.* [15], wherein the PAM-specific amidase was capable of hydrolyzing other short-chain aliphatic amides.

According to the results of degradation analysis, we verified that the HI47 strain utilized PAM in aerobic conditions. However, PAM could not be completely degraded in our experiments. Even when we supplemented the polymer medium with sufficient glucose during continuous cultivation, the PAM removal efficiency did not exceed 85%. Other factors like chemical substances, thermal and mechanical effects might influence PAM degradation rate. Intermolecular cross-linking and bridging of polymer also affects its degradation. However, we did not find any extracellular enzymes relate to the breaking of carbon–carbon bonds in the experiment. Perhaps the cleavage is associated with constitutive enzymes or proteins. Further studies need to be conducted to explore the mechanism of breaking of PAM backbone chain.

## 4. Conclusions

In this work, A PAM-degrading bacterial strain *P. putida* HI47 was isolated from dewatered sludge. The bacterial isolates grew well on medium with PAM as the sole source of nutrients at pH 7.2–7.6 and 36 °C–42 °C. The presence of glucose improved bacterial growth and the rate of degradation. According to the GFC, FT-IR, HPLC, SDS-PAGE, and *N*-terminal sequencing analyses, we found PAM was partly cleaved into small molecular oligomer derivatives and the part of amide groups were converted into carboxyl groups by a PAM-induced extracellular enzyme from the aliphatic amidase family. PAM biodegradation did not accumulate acrylamide monomers. The results indicated that the HI47 strain is a potential candidate for safe PAM degradation. However, in present work we mainly focus on characterization of the strain and ignore its actual performance in dewatered sludge. It still remains to be investigated whether the performance of HI47 in sludge is the same as it in laboratory experiment. Future works are needed to explore the PAM degradation effect of strain in sludge application.

## Appendix

**Table A1:** Biochemical characteristics of strain HI47 identified by using GN card in VITEK 2 Compact.

Test	Representation	Result
APPA	Ala-Phe-Pro-ARYLAMIDASE	−
ADO	ADONITOL	−
PyrA	l-Pyrrolydonyl-ARYLAMIDASE	−
lARL	l-ARABITOL	−
dCEL	d-CELLOBIOSE	−
BGAL	β-GALACTOSIDASE	−
H2S	H_2_S PRODUCTION	−
BNAG	β-ACETYL-GLUCOSAMINIDASE	−
AGLTp	Glutamyl arylamidase pNA	+
dGLU	d-GLUCOSE	+
GGT	γ-GLUTAMYL-TRANSFERASE	+
OFF	FERMENTATION/GLUCOSE	−
BGLU	β-GLUCOSIDASE	−
dMAL	d-MALTOSE	−
dMAN	d-MANNITOL	+
dMNE	d-MANNOSE	+
BXYL	β-XYLOSIDASE	−
BAlap	β-Alanine arylamidase pNA	+
ProA	l-Proline ARYLAMIDASE	+
LIP	LIPASE	−
PLE	PALATINOSE	−
TyrA	Tyrosine ARYLAMIDASE	+
URE	UREASE	+
dSOR	d-SORBITOL	−
SAC	SACCHAROSE/SUCROSE	−
dTAG	d-TAGATOSE	−
dTRE	d-TRHALOSE	−
CIT	CITRATE (SODIUM)	+
MNT	MALONATE	+
5KG	5-KETO-d-GLUCONATE	−
lLATk	l-LACTATE alkalinisation	+
AGLU	α-GLUCOSIDASE	−
SUCT	SUCCINATE alkalinisation	+
NAGA	BETA-N-NCETYL-GALACTOSAMINIDASE	−
AGAL	ALPHA-GALACTOSIDASE	−
PHOS	PHOSPHATASE	−
GlyA	Glycine ARYLAMIDASE	−
ODC	ORNITHINE DECARBOXYLASE	−
LDC	LYSINE DECARBOXYLASE	−
lHISa	l-HISTIDINE assimilation	−
CMT	COUMARATE	+
BGUR	β-GLUCORONIDASE	−
O129R	O/129 RESISTANCE (comp.vibrio)	+
GGAA	Glu-Gyl-Arg-ARYLAMIDASE	+
lMLTa	l-MALATE assimilation	+
ELLM	ELLMAN	−
lLATa	l-LACTATE assimilation	−
